# N-acetylcysteine supplementation did not reverse mitochondrial oxidative stress, apoptosis, and inflammation in the salivary glands of hyperglycemic rats

**DOI:** 10.1038/s41387-021-00177-w

**Published:** 2021-11-09

**Authors:** Zalewska Anna, Kuć Joanna, Zięba Sara, Matczuk Jan, Kostecka-Sochoń Paula, Szarmach Izabela, Ładny Jerzy Robert, Żendzian-Piotrowska Małgorzata, Maciejczyk Mateusz

**Affiliations:** 1 Independent Laboratory of Experimental Dentistry, Medical University of Bialystok, Bialystok, Poland; 2 Department of Restorative Dentistry, Medical University of Bialystok, Bialystok, Poland; 3 Department of Prosthodontics, Medical University of Bialystok, Bialystok, Poland; 4 Doctoral Studies, Medical University of Bialystok, Bialystok, Poland; 5 County Veterinary Inspection, Bialystok, Poland; 6 Department of Orthodontics, Medical University of Bialystok, Bialystok, Poland; 7 Department of Emergency Medicine and Disasters, Medical University of Bialystok, Bialystok, Poland; 8 Department of Hygiene, Epidemiology and Ergonomics, Medical University of Bialystok, Bialystok, Poland

**Keywords:** Enzymes, Cardiovascular diseases

## Abstract

**Background/objectives:**

Previous studies have shown that N-acetylcysteine (NAC) supplementation with the simultaneous inclusion of HFD prevents salivary glands from oxidative stress and mitochondrial dysfunction. In this experiment, we examined if NAC supplementation could reverse the harmful effect of HFD on mitochondrial function, reduce the severity of apoptosis, and the activity of pro-oxidative enzymes in the salivary glands of rats with confirmed hyperglycemia.

**Subjects/methods:**

Wistar rats were fed the standard or high-fat (HFD) diet for 10 weeks. After 6 weeks of the experiment, HFD rats were diagnosed with hyperglycemia and for the next 4 weeks, the animals were given NAC intragastrically. In the mitochondrial fraction of the parotid (PG) and submandibular salivary glands (SMG), we assessed redox status, inflammation, and apoptosis.

**Results:**

The inclusion of NAC increased the activity of mitochondrial complexes I and II + III as well as decreased the concentration of interleukin-1β, tumor necrosis factor α, and caspase-3, but only in the parotid glands of rats with hyperglycemia compared to the HFD group. However, N-acetylcysteine supplementation did not reduce the activity of caspase-9 or the Bax/Bcl-2 ratio in PG and SMG mitochondria. In both salivary glands we observed reduced activity of cytochrome c oxidase, NADPH oxidase, and xanthine oxidase, as well as hindered production of ROS and lower ADP/ATP radio, but the levels of these parameters were not comparable to the control group.

**Conclusions:**

We demonstrated that NAC supplementation restores the glutathione ratio only in the mitochondria of the submandibular salivary glands. The supply of NAC did not significantly affect the other measured parameters. Our results indicate that NAC supplementation provides little protection against free radicals, apoptosis, and inflammation in the salivary gland mitochondria of HFD rats. Stimulated salivary secretion in hyperglycaemic rats supplemented with NAC seems to be closely related to mitochondrial respiratory capacity and appropriate ATP level.

## Introduction

Chronic exposure to the high-fat diet (HFD) not only interferes with the body’s carbohydrate metabolism but also contributes to the dysregulation of mitochondrial activity [1–3]. Our previous research found that HFD disrupts the function of the mitochondria, decreases ATP production, and enhances reactive oxygen species (ROS) formation in the salivary glands [2]. Hyperglycemia results in greater oxygen consumption in the mitochondria, which is inextricably linked with ROS overproduction [4] and boosts the redox potential by shifting the oxygen transport towards complex II of the respiratory chain [5]. Hyperglycemia is accompanied by an increased rate of ADP regeneration, with reduced ATP production and increased depolarization of mitochondrial membranes [6]. It is believed that hyperglycemia-related mitochondrial dysfunction leads to pathological changes that promote insulin resistance (IR) as well as type 2 diabetes and the associated organ complications [7]. Considering the above, it appears reasonable to include substances that would not only restore normal glycemia and tissue sensitivity to insulin but also positively affect mitochondrial functions at the stage of hyperglycemia. Studies have proved that N-acetylcysteine (NAC)—an analog of reduced glutathione (GSH)—may enhance the functioning of mitochondria, especially oxidative phosphorylation [8]. NAC improves the activity of the mitochondrial complexes (I and IV) in the liver [9], completely restores the function of the complex II in the heart mitochondria and partially complex I in the mitochondria of the brain of aging rats [10]. The administration of NAC up to 2 h after the brain injury resulted in an improvement in the activity of mitochondrial complexes, enhanced oxidative phosphorylation, and a reduction in the rate of ROS production [11]. According to the authors, the restoration of mitochondrial function resulted from an increase in disulfide bonds of protein chains of the mitochondrial enzymes.

HFD is becoming more and more common in Western societies. Therefore, measures should be taken to prevent/reverse its harmful health effects. Our earlier study demonstrated that the simultaneous inclusion of HFD and NAC supplementation prevents hyperglycemia, improved the cytoplasmic antioxidant barrier of both salivary glands, and protected against oxidative damage in the parotid glands of IR rats [12]. This model of NAC supplementation leads to a significant increase in the activity of complexes I and II + III as well as cytochrome c oxidase (COX) and reduces the ADP/ATP ratio in the salivary glands of rats compared to rats fed the HFD only to the level observed in the control group, which is noteworthy [8]. However, there have been no studies to evaluate the effect of NAC administration on mitochondrial function in the salivary glands of rats after hyperglycemic state induction in the so-called pre-diabetes condition. In other words, it was not assessed whether NAC could reverse the harmful effect of HFD on mitochondrial function.

Therefore, in the presented study, we decided to evaluate whether NAC supplementation could reverse the mitochondrial chain function, reduce the severity of apoptosis, and the activity of the mitochondrial prooxidative enzymes in the salivary glands of rats with HFD-induced hyperglycemia. We also tried to answer the question if the salivary gland dysfunction observed in hyperglycaemic rats supplemented by NAC has a relationship with mitochondrial dysfunction.

## Materials and methods

The study was conducted after prior approval of the Local Ethical Committee for Animal Experiments in Olsztyn (No. 21/2017). All experimental procedures were planned by three qualified and experienced persons (A.Z., M.M., and M.Ż.-P.). The practical implementation of the experiment was carried out by two trained and experienced scientists (M.M. and J.M.).

Throughout the experiment, male Wistar rats (*Rattus norvegicus*; *n* = 40; initial body weight (BW) = 69–72 g; initial age = 4–5 weeks) were grown in one laboratory room (2 individuals per cage). They were kept under the following conditions: 12 h of light/12 h of darkness; 20–21 °C; humidity dependent on external conditions. The rats consumed drinking water without restriction. The period of acclimatization to environmental conditions was 5 days. Then, the rodents were divided randomly into 2 equal groups (*n* = 20):

- Group 1—fed the standard diet (Research Diet, USA, catalog number: D12450J): 10% fats, 20% proteins, 70% carbohydrates.
- Group 2—fed the high-fat diet (Research Diet, USA, catalog number: D12492) containing 60% fats, 20% proteins, and 20% carbohydrates.

The above procedure was maintained for 6 weeks. The composition of diets was presented in Table 1. However, blinding was not used in our study.

**Table 1:** General composition of the control and high-fat diet (HFD).

	Ingredient	Control diet (g)	HFD (g)
Carbohydrate	Starch, corn	506.20	0.00
	Lodex 10	125.00	125.00
	Sucrose, fine granulated	72.80	72.80
Fat	Soybean oil, USP	25.00	25.00
	Lard	20.00	245.00
Protein	Casein, lactic, 30 mesh	200.00	200.00
	Cystine, L	3.00	3.00
Fiber	Solka floc, FCC200	50.00	50.00
Mineral	S10026B	50.00	50.00
Vitamins	Choline bitartrate	2.00	2.00
	V10001C	1.00	1.00

After 6 weeks, all rats of both groups had the concentration of glucose in venous blood obtained from the tail vein determined. The criteria excluding from further studies were: blood glucose concentration below 70 mg/dL and above 150 mg/dL and poor general condition (lethargy, lack of appetite, or any signs of illness). The blood glucose concentration in all rats fed the high-fat diet was above 95 mg/dL and below 135 mg/dL, and below 86 mg/dL in group 1. All rats were also in good general condition.

Then, both groups were randomly divided into 2 subgroups of 10 individuals each. The following scheme was implemented for the next 4 weeks:

- Group C—fed the standard diet (Research Diet, USA, catalog number: D12450J): 10% fats, 20% proteins, 70% carbohydrates.
- C + NAC group—fed the standard diet (Research Diet, USA, catalog number: D12450J): 10% fats, 20% proteins, 70% carbohydrates as well as had NAC solution (500 mg/kg B.W., Sigma-Aldrich catalog number A9165) administered intragastrically once a day (between 8 AM and 9 AM).
- HFD group—fed the high-fat diet (Research Diet, USA, catalog number D12492) containing 60% fats, 20% proteins, and 20% carbohydrates.
- HFD + NAC group—fed the high-fat diet (Research Diet, USA, catalog number D12492) containing 60% fats, 20% proteins, and 20% carbohydrates, and had NAC solution (500 mg/kg B.W., Sigma-Aldrich catalog number A9165) administered intragastrically once a day (between 8 AM and 9 AM).

To maintain identical procedures in groups C and HFD, saline solution was administered intragastrically once a day at a volume of 2 mL/kg BW.

Both the saline solution and NAC were administered always by two qualified specialists (J.M. and M.M.). Food consumption was assessed every day in the morning. The NAC dose was estimated every two days, which was preceded by the BW evaluation of the rats.

The dose of NAC (500 mg/kg BW) was selected after a thorough literature search. The dose implemented in our study is safe for animals, does not introduce toxic influence, and has a beneficial antioxidant effect [8, 13].

All rats in the four groups at the end of the experiment were in good general condition. Therefore, all rats underwent the last stage of the experiment: sacrifice and tissue removal.

Four weeks after the introduction of NAC supplementation (10 weeks from the beginning of HFD administration), after overnight starvation, the rats were dosed with phenobarbital (80 mg/kg BW) and then placed on a heated table. A tail vein was taken to measure glucose concentration. The criterion excluding from further studies was blood glucose concentration below 70 mg/dL and above 150 mg/dL. The rate of stimulated and non-stimulated saliva secretion was measured, as described in our earlier study [8]. After cutting the abdominal integuments, blood was collected from the abdominal aorta into a tube with EDTA. The obtained plasma was enriched with antiprotease solution (1:10, protease inhibitors) and BHT (Sigma-Aldrich, Germany). Totally, 100 µL of the collected plasma was used to assay insulin concentration (ELISA kit, Shibayagi Co., Gunma, Japan); the samples were placed in liquid nitrogen and stored at −80 °C. The content of plasma-free fatty acids was analyzed by gas chromatography [14]. This determination was performed within 2 weeks after the plasma samples were frozen.

Tissue sensitivity to insulin was assayed using the homeostasis model assessment of IR: HOMA-IR index = (fasting insulin [U/mL] × fasting glucose [mM]/22.5) [15].

The salivary gland index was calculated by dividing the weight of salivary glands (parotid or submandibular) by total BW.

In the next stage, the submandibular and parotid salivary glands were collected by M.M. and J.M. and treated on the same day. The harvested tissues were cleaned, weighed, and prepared for homogenization in ice-cold isolation buffer (1:10, w/v) using Teflon-and-glass homogenizer. The composition of mitochondrial isolation buffer was as follows: 250 mM saccharose, 5 mM Tris (Tris(hydroxymethyl)aminomethane)-HCl, and 2 mM ethylene glycol bis(2-aminoethyl)tetraacetic acid (EGTA), pH 7.4. Antiprotease solution (1:10, protease inhibitors) and BHT (Sigma-Aldrich, Germany) were also added to the buffer. The homogenates were spun (500×*g*, 10 min, 4 °C), followed by two more spins at 8000×*g* [16]. An isolating buffer was added to the mitochondrial sediment and the procedure was continued on the same day. The assessment of the purity of the mitochondrial fraction was made on the basis of western blot analysis. This analysis excludes the presence of cytoplasmic glyceraldehyde 3-phosphate dehydrogenase or the nuclear marker histone H3 [8].

### Biochemical determinations

In the obtained mitochondrial fraction, we assayed the activity of mitochondrial membrane protein complexes, the ADP/ATP ratio, hydrogen peroxide production, the activity of mitochondrial citrate synthase (CS), cytochrome c oxidase (COX), NADPH oxidase (NOX), xanthine oxidase (XO), free radical production—by means of the DCFH-DA test, caspase-3 and -9 activity, the concentration of Bax, Bcl-2, interleukin 1 (IL-1β), the content of reduced glutathione (GSH), oxidized glutathione (GSSG), and redox state. The obtained results were standardized to 1 mg of total mitochondrial proteins.

### Mitochondrial membrane protein complexes

The activity of mitochondrial complex I (ubiquinone oxidoreductase; E.C. 1.6.5.3) was analyzed colorimetrically in duplicate samples based on 2,6-dichloroindophenol (DCIP) reduction by electrons accepted from ubiquinone-1,2,3-Dimethoxy-5-methyl-6-(3-methyl-2-butenyl)-1,4-benzoquinone (coenzyme Q1) [17].

The activities of mitochondrial complex II (succinate dehydrogenase; E.C. 1.3.5.1) and complex II + III (succinate dehydrogenase + quinol-cytochrome-c reductase, E.C. 1.10.2.2) were assayed colorimetrically in duplicate samples according to Rustin et al. [18].

### ADP/ATP ratio and H_2_O_2_ level

The ADP/ATP ratio in the salivary gland mitochondria was analyzed in duplicate samples according to the bioluminescent method based on the conversion of ATP to ADP by luciferase. The procedure was performed using a commercial kit (ADP/ATP Ratio Assay Kit ab65313; Abcam, USA) according to the manufacturer’s instructions.

Mitochondrial hydrogen peroxide (H_2_O_2_) was measured in duplicate samples using the Amplex Red fluorimetric method. H_2_O_2_ level was calculated using a standard curve of H_2_O_2_ stabilized solution [19].

### Activity of COX

Mitochondrial COX, complex IV, E.C. 1.9.3.1 activity was determined in duplicate samples using the colorimetric method. Oxidation of reduced cytochrome c was measured at 550 nm wavelength [20].

### Activity of CS

Mitochondrial CS, E.C. 2.3.3.1 activity was estimated colorimetrically in duplicate samples using enzymatic reaction with 5-thio-2-nitrobenzoic acid (TNB) formed from Ellman’s reagent (DTNB, 5,5′-dithiobis-(2-nitrobenzoic acid) [21].

### ROS-generating enzymes

Mitochondrial NOX, E.C. 1.6.3.1 activity was established in duplicate samples by a luminescence method. One unit of NOX activity was determined as the quantity of the enzyme required to release 1 nM of the superoxide anion for 1 min [22].

Mitochondrial XO, E.C. 1.17.3.2. the activity was established in duplicate samples based on the uric acid formation. An increase in uric acid optical density was measured at 290 nm wavelength. One unit of XO activity was determined as the quantity of the enzyme required to release 1 μM of uric acid for 1 min [23].

Mitochondrial production of ROS was analyzed spectrofluorimetrically in duplicate samples using 2,7-dichlorodihydrofluorescein diacetate (DCFH-DA). In this assay, DCFH-DA was de-esterified to 2,7-dichlorodihydrofluorescein (DCFH) [24].

Mitochondrial ROS-generating enzymes were measured immediately after salivary gland collection [25].

### Inflammation and apoptosis

The activity of mitochondrial caspase-3 (CAS-3, EC 3.4.22.56) was estimated colorimetrically in duplicate samples using Ac-DEVD-pNA (Ac-Asp-Glu-Val-Asp-p-nitroanilide; Km = 9.7 µM) as a substrate. The quantity of p-nitroaniline (p-NA) released by CAS-3 activity was analyzed at a 405 nm wavelength [26].

The activity of mitochondrial caspase-9 (CAS-9, EC 3.4.22.62) was analyzed fluorometrically in duplicate samples using AFC:7-amino-4-(trifluoromethyl)coumarin as a substrate. After cleavage of the substrate by CAS-9, free AFC emitted yellow and green fluorescence (505 nm wavelength) which was quantified using a fluorometer.

The levels of mitochondrial Bax, Bcl-2, and tumor necrosis factor α (TNF-α) were established colorimetrically in duplicate samples by commercial ELISA kits (Rat Apoptosis regulator BAX and rat apoptosis regulator Bcl-2, rat tumor necrosis factor, all from EIAab (Wuhan, China).

The level of mitochondrial interleukin-1β (IL-1β) was measured in duplicate samples using a commercial enzyme-linked immunosorbent assay (ELISA) kit (IL-1β ELISA kit, R&D Systems; Minneapolis, USA) according to the manufacturer’s instructions.

Inflammation and apoptosis parameters were also determined by real-time polymerase chain reaction (RT-PCR) in whole salivary gland samples. For RNA purification, frozen fragments of left salivary glands were homogenized by grinding with liquid nitrogen and dissolved in RTK Buffer provided in the Qiagen RNeasy Mini Kit (Qiagen, Hilden, Germany), according to the manufacturer’s instructions. The RNA concentration and purity were checked at 260 nm and 280 nm using Infinite M200 PRO Multimode Microplate Reader (Tecan). The mRNA expression levels of *IL-1β*, *CAS-3*, and *CAS-9* were analyzed using the LightCycler^®^ 96 Real-Time PCR System (Mannheim Germany) with SYBR Green Supermix (Bio-Rad Laboratories, Hercules, CA), according to the manufacturer’s instructions. Duplicate plates were tested for each condition. Briefly, the three steps of the PCR included: denaturation at 94 °C for 2 min, annealing at 62 °C for 30 s with fluorescence reading, and extension at 72 °C for 30 s. β2-microglobulin (B2M) was used as a housekeeping gene. Gene expression was assessed by measurement of steady-state levels of mRNA. Relative expression from RT-PCR was calculated from the equation 2A-B/2C-D (where A = Cycle Threshold [Ct] number for the gene of interest in the first control sample; B = Ct number for the gene of interest in the analyzed sample; C = Ct number for the housekeeping gene in the first control sample; D = Ct number for housekeeping gene in the analyzed sample). Sequences of PCR primers are shown (*IL-1β* forward AAACAGATGAAGTGCTCCTTCCAGG and revers TGGAGAACACCACTTGTTGCTCCA; *CAS-3* forward 5′-TGCAGCATGCTGAAGCTGTA and revers 5′-GAGCATGGACACAATACACG; *CAS-9* forward 5′-AGCAGAGAGTAGTGAAGCTG and revers 5′-ACACAGACATCATGAGCTCC; *B2M* forward 5′-AAGTATACTCACGCCACCCA and revers 5′-AAGACCAGTCCTTGCTGAAG).

### Reduced and oxidized glutathione content and redox status

The level of mitochondrial total glutathione was measured in triplicate samples based on an enzymatic reaction with glutathione reductase (GR), DTNB, and NADPH [27]. Before the determination of mitochondrial oxidized glutathione (GSSG), the samples had been thawed and neutralized to pH 6–7. For this purpose, 1 M triethanolamine hydrochloride (TEA) was used. Then, the samples were incubated with 2-ethenylpyridine (2-vinylpyridine) [27]. After that, measures were estimated in a manner similar to the assay performed for mitochondrial total glutathione. The level of mitochondrial reduced glutathione (GSH) was calculated using the formula: [mitochondrial GSH] = [mitochondrial total glutathione] − [mitochondrial GSSG] [27].

The mitochondrial redox (oxidation/reduction) status was calculated using the formula: [GSH]^2^/[GSSG] [16].

### Enzymatic antioxidants and oxidative stress

The activity of mitochondrial Cu–Zn–superoxide dismutase (SOD, EC 1.15.1.1) was determined in duplicate samples by a spectrophotometric method. For this purpose, the inhibition rate of adrenaline oxidation to adrenochrome was measured [28]. It was qualified that one unit of enzyme activity inhibits the oxidation of adrenaline by 50% at 480 nm.

Mitochondrial sialoperoxidase (Px, E.C. 1.11.1.7) activity was determined colorimetrically in duplicate samples based on the reduction of DTNB to TNB [29]. A decrease in the absorbance of TNB at 412 nm was measured five times with intervals of 30 s.

The activity of serum glutathione peroxidase (GPx, E.C. 1.11.1.9) was assessed in duplicate samples by a colorimetric method at 340 nm. For this purpose, the reduction of organic peroxides in the presence of NADPH was measured [27]. It was assumed that one unit of enzyme activity catalyzes the oxidation of 1 μM NADPH per 1 min.

Mitochondrial catalase (CAT, E.C. 1.11.1.6) activity was analyzed in duplicate samples by measuring the rate of hydrogen peroxide decomposition. For this purpose, the colorimetric method at a wavelength of 240 nm was used [30]. It was assumed that one unit of enzyme activity decomposes 1 mM of hydrogen peroxide per 1 min.

Mitochondrial malondialdehyde (MDA) concentration was analyzed in duplicate samples by measuring thiobarbituric acid reactive substances (TBARS). 1,1,3,3-tetramethoxypropane was used as a standard [31].

### Histological studies

The left submandibular and parotid glands were fixed with 10% phosphate-buffered formaldehyde and processed for histology using standard techniques. Tissues were embedded in paraffin for making five-micron sections, deparaffinized, rehydrated, and stained with hematoxylin–eosin (H–E) as previously described [32]. Then, the slides were examined under a light microscope (OPLYMPUS BX 51, OLYMPUS; 60× magnification). Cell morphology and a number of fat vacuoles were evaluated according to the following criteria: +: single vacuoles in the cytoplasm of salivary acinar cells; ++: 5–10% of each section occupied by pathological alternations; +++: 11–20% of each section occupied by pathological alternations; ++++: 21–30% of each section occupied by pathological alternations; +++++: >30% of each section occupied by pathological alternations [32]. Histological studies were carried out by an experienced histologist.

### Statistical analysis

The results of the conducted study were then subjected to statistical analysis, determining mean values, standard deviations for a given group, and one-way analysis of variance (one-way ANOVA). The significance of the differences between the mean values for the groups was determined based on Tukey’s post hoc. Normality of the distribution was confirmed by the Kolmogorov–Smirnov test, while homogeneity of variance was assumed based on Levene’s test. Correlations between the assayed parameters were assessed using the Pearson correlation coefficient. The value of *p* < 0.05 was considered statistically significant. The following levels of statistical significance were used: *p < 0.05, **p < 0.005, ***p < 0.0005, ****p < 0.0001.

The number of animals was calculated a priori based on previous preliminary experiments. Type I error *α* = 0.05, and statistical power (type II error) of 0.9 were considered. The minimum number of rats in one group was eight. Thus, the analysis was performed on ten individuals. The analysis was conducted using the statistical package GraphPad Prism 8.3.0 for MacOS (GraphPad Software, La Jolla, CA, USA).

## Results

The results showed a normal distribution, and the variances in the compared groups were similar to each other.

### General characteristics and salivary gland function

#### Before NAC administration

After 4 weeks of the experiment, the bodyweight of rats fed an HFD diet was significantly higher (C: 285.8 ± 17.54, HFD: 350.4 ± 10.32, *p* < 0.0001), while food intake was significantly lower compared to the control group (C: 17.68 ± 3.1, HFD: 12.43 ± 1.82, *p* < 0.0001). Hyperglycemia was confirmed in all rats fed the HFD diet (C: 95.63 ± 6.15, HFD: 158.1 ± 8.18, *p* < 0.0001).

#### After NAC administration

**Fig. 1:**
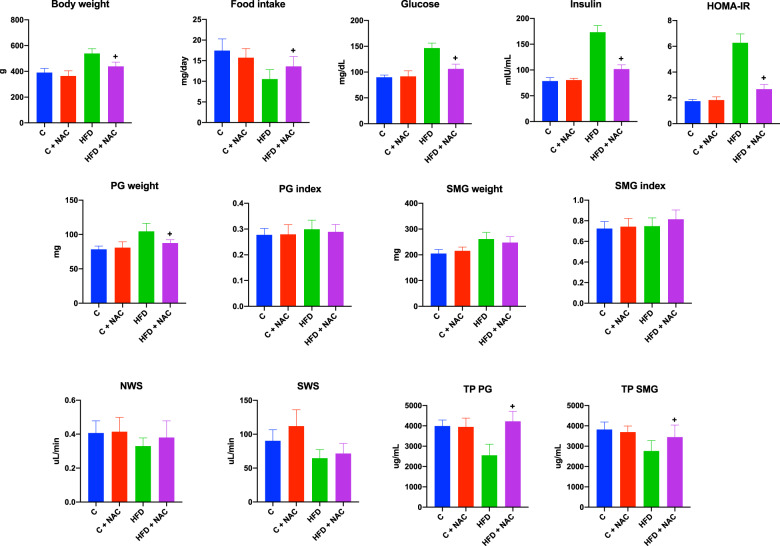
General characteristics of rats and salivary gland function. ^a^*p* < 0.05 vs. C, ^b^*p* < 0.05 vs. HFD. C control group, C + NAC control rats + NAC administration, HFD high-fat-fed group, HFD + NAC high-fat-fed rats + NAC administration, HOMA-IR homeostasis model assessment of insulin resistance, NWS non-stimulated whole salivary flow PG parotid salivary glands, SMG submandibular salivary glands, SWS stimulated whole salivary flow, TP total salivary proteins.

Despite significantly lower food intake, rats fed a high-fat diet had significantly higher body weight than the control group. The HFD rats administered with NAC had significantly higher food intake and statistically lower body weight than the HFD group. Glycemia, insulinemia, and HOMA-IR levels in the HFD group were significantly higher than the control and HFD + NAC groups. We did not notice any differences in the assessed parameters between groups C and C + NAC or C and HFD + NAC. The PG and SMG weight of rats fed the high-fat diet was significantly higher than the control group. Only the parotid gland’s mass decreased after the introduction of NAC administration. The non-stimulated salivary secretion did not differ significantly between the groups, while the stimulated salivation in the HFD rats was significantly lower than the control group. NAC administration did not affect the stimulated salivation and was significantly lower than the control group. The total protein content in the parotid and submandibular salivary gland homogenate of rats fed a high-fat diet was significantly reduced compared to the control group. After NAC administration, the total protein content in the homogenate of the PG and SMG in the rodents from the HFD + NAC group was significantly higher compared to the rats fed HFD (Fig. 1).

### Mitochondrial chain

#### Parotid glands

**Fig. 2:**
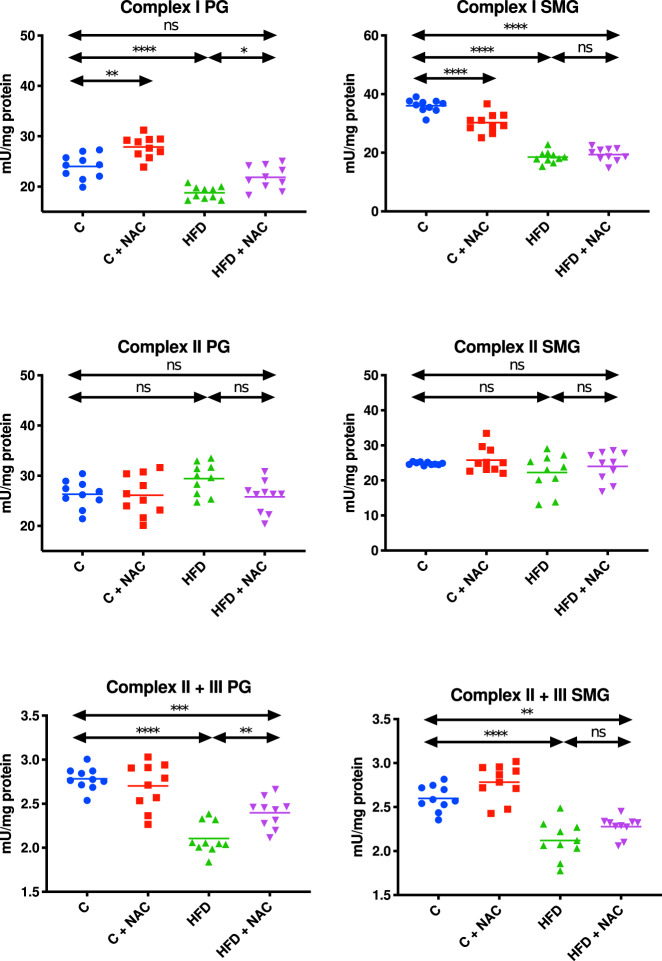
Impact of N-acetylcysteine (NAC) administration on mitochondrial respiratory complexes in the parotid and submandibular glands of rats. *p < 0.05, **p < 0.005, ***p < 0.0005, ****p < 0.0001. C control rats, C + NAC control rats + NAC administration, HFD rats fed the high-fat diet, HFD + NAC rats fed the high-fat diet + NAC administration, ns not significant, PG parotid salivary glands, SMG submandibular salivary glands.

The activity of complex I in the mitochondrial fraction of the parotid glands of HFD rats was significantly lower compared to the control group (↓22% *p* < 0.0001). The introduction of NAC administration in hyperglycemic rats resulted in considerably higher mitochondrial complex I activity compared to the group fed the high-fat diet (↑14% *p* = 0.0132). Moreover, the activity of this complex in the HFD + NAC group was at a similar level as in the control group. Interestingly, NAC administration in the group of rats not provided with any specific diet resulted in significantly increased activity of mitochondrial complex I compared to the control rats without administration (↑14% *p* = 0.0011) (Fig. 2).

Complex II activity in the mitochondrial fraction of the parotid glands was comparable in all groups of rats (Fig. 2).

The activity of mitochondrial complexes II + III in the parotid glands of HFD rodents was significantly lower in comparison with the control group (↓24% *p* < 0.0001). The inclusion of NAC administration in rats with hyperglycemia significantly increased the activity of mitochondrial complex II + III compared to the group fed the high-fat diet (↑12% *p* = 0.0081), but it remained lower than in the control group (↓14% *p* = 0.0003) (Fig. 2).

#### Submandibular glands

The activity of complexes I and II + III in the mitochondrial fraction of the submandibular salivary glands of HFD rats was significantly lower compared to the control group (↓49% *p* < 0.0001, ↓23% *p* < 0.0001, respectively). The introduction of NAC s administration in rats with hyperglycemia did not entail any significant changes in the activity of both mitochondrial complexes compared to the HFD group. Furthermore, the activity of these complexes in the HFD + NAC group remained lower compared to the activity of complexes I and II + III in the control group (↓46% *p* < 0.0001, ↓12% *p* = 0.001, respectively). Interestingly, NAC administration in the group of rats without a particular diet resulted in a considerable decrease in mitochondrial complex I activity compared to the controls without NAC administration (↓16% *p* < 0.0001) (Fig. 2).

The activity of complex II in the mitochondrial fraction of the submandibular glands was comparable in all groups of rats (Fig. 2).

### ADP/ATP ratio

**Fig. 3:**
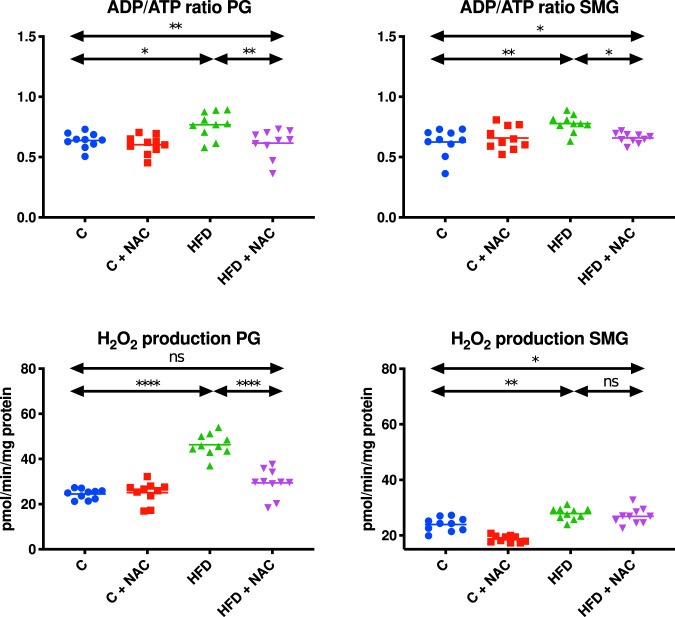
Impact of N-acetylcysteine (NAC) administration on the ADP/ATP ratio and hydrogen peroxide production in the salivary gland mitochondria. *p < 0.05, **p < 0.005, ****p < 0.0001. C control rats, C + NAC control rats + NAC administration, HFD rats fed the high-fat diet, HFD + NAC rats fed the high-fat diet + NAC administration, H_2_O_2_ hydrogen peroxide, ns not significant, PG parotid salivary glands, SMG submandibular salivary glands.

The ADP/ATP ratio in the mitochondria of the parotid and submandibular salivary glands of HFD rats was significantly higher compared to the control group (↑17% *p* = 0.0193, ↑20% *p* = 0.0017, respectively). The inclusion of NAC administration in rats with hyperglycemia resulted in a decrease in the ADP/ATP ratio in the mitochondria of the parotid and submandibular glands compared to the HFD group (↓20% *p* = 0.005, ↓16% *p* = 0.0165, respectively) (Fig. 3).

### H_2_O_2_ concentration

The concentration of H_2_O_2_ in the mitochondrial fraction of the parotid and submandibular salivary glands of rats fed the HFD was significantly higher compared to the control group (↑47% *p* < 0.0001, ↑14% *p* = 0.0025, respectively). The introduction of NAC administration in hyperglycemic rats resulted in decreased concentration of H_2_O_2_ in the mitochondria of the parotid glands compared to the HFD group (↓36% *p* < 0.0001). Moreover, mitochondrial H_2_O_2_ concentration in the HFD + NAC group was at a level comparable to the controls. The inclusion of NAC administration in rats with hyperglycemia did not cause any significant changes in mitochondrial H_2_O_2_ concentration in the submandibular glands compared to the HFD group, but it was significantly higher compared to the control group (↑11% *p* = 0.0351) (Fig. 3).

### CS activity

**Fig. 4:**
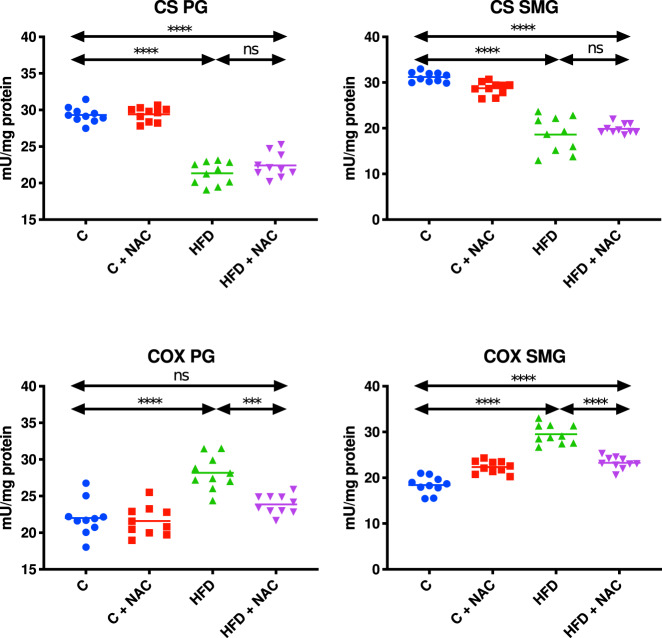
Impact of N-acetylcysteine (NAC) administration on citrate synthase and cytochrome c oxidase activity in the salivary gland mitochondria. ***p < 0.0005, ****p < 0.0001. C control rats, C + NAC control rats + NAC administration, COX cytochrome c oxidase, CS citrate synthase, HFD rats fed the high-fat diet, HFD + NAC rats fed the high-fat diet + NAC administration, ns not significant, PG parotid salivary glands, SMG submandibular salivary glands.

The activity of CS in the mitochondrial fraction of the parotid and submandibular salivary glands of HFD rats was significantly lower compared to the control group (↓27% *p* < 0.0001, ↓40% p < 0.0001, respectively). The inclusion of NAC administration in rats with hyperglycemia did not cause any considerable changes in mitochondrial CS activity compared to the HFD group. What is more, the activity of this enzyme in the parotid and submandibular glands of the HFD + NAC group remained lower compared to mitochondrial CS activity in the control group (↓24% *p* < 0.0001, ↓36% *p* < 0.0001, respectively) (Fig. 4).

The COX activity in the mitochondrial fraction of the parotid salivary glands of HFD rats was significantly higher compared to the control group (↑22 % p < 0.0001). NAC administration of rats with hyperglycemia remaining on the high-fat diet resulted in lower mitochondrial COX activity compared to the HFD group (↓15% *p* = 0.0002). Interestingly, mitochondrial COX activity in the HFD + NAC group was at a similar level to that observed in the control group (Fig. 4).

The activity of COX in the mitochondrial fraction of the submandibular salivary glands of HFD rats was significantly enhanced compared to the control group (↑38% *p* < 0.0001). NAC administration of hyperglycemic rats fed the HFD resulted in decreased mitochondrial COX activity compared to the HFD group (↓21% *p* < 0.0001). Interestingly, mitochondrial COX activity in the HFD + NAC group was significantly higher compared to that of the control group (↑21% *p* < 0.0001) (Fig. 4).

### The activity of ROS-generating enzymes and DCFH-DA

**Fig. 5:**
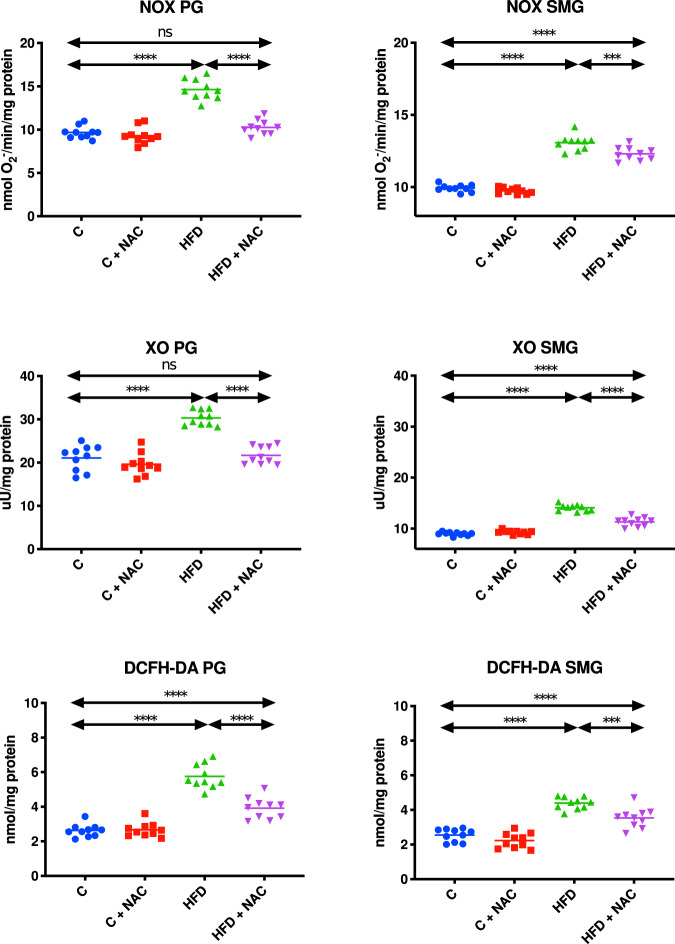
Impact of N-acetylcysteine (NAC) administration on pro-oxidant enzymes and free radical production in the salivary gland mitochondria. ***p < 0.0005, ****p < 0.0001. C control rats, C + NAC control rats + NAC administration, DCFH-DA 2,7-dichlorodihydrofluorescein diacetate, HFD rats fed the high-fat diet, HFD + NAC rats fed the high-fat diet + NAC administration, NOX NADPH oxidase, ns not significant, PG parotid salivary glands, SMG submandibular salivary glands, XO xanthine oxidase.

NOX and XO activity in the mitochondrial fraction of the parotid (↑34% *p* < 0.0001, ↑30% *p* < 0.0001, respectively) and submandibular (↑24% *p* < 0.0001, ↑36% *p* < 0.0001, respectively) glands of HFD rats was significantly higher compared to the control group. The inclusion of NAC administration in hyperglycemic rats resulted in considerably lower mitochondrial NOX and XO activity in the parotid (↓30% *p* < 0.0001, ↓28% *p* < 0.0001, respectively) and submandibular (↓6% *p* = 0.0004, ↓20% *p* < 0.0001, respectively) glands compared to the group fed the high-fat diet. However, the activity of mitochondrial NOX and XO in the submandibular glands of HFD rats administered with NAC remained significantly higher compared to the control rats (↑19% *p* < 0.0001, ↑21% *p* < 0.0001, respectively). The activity of NOX and XO in the mitochondria of the parotid salivary glands of HFD + NAC rats was at a similar level to that in the control group (Fig. 5).

DCFH-DA in the mitochondrial fraction in both the parotid and submandibular glands of HFD rats was significantly higher compared to the control group (↑54% *p* < 0.0001, ↑42% *p* < 0.0001, respectively). The 4-week NAC administration introduced at the hyperglycemic stage resulted in considerably lower mitochondrial DCFH-DA content in the parotid as well as submandibular glands compared to the group fed the HFD exclusively (↓32% *p* < 0.0001, ↓18% *p* = 0.0005, respectively). However, it should be emphasized that NAC administration did not effectively prevent the increase of mitochondrial DCFH-DA in both salivary glands of rats since its level in both the parotid and submandibular glands of HFD + NAC rats was significantly higher compared to the control group (↑33% *p* < 0.0001, ↑28% *p* < 0.0001, respectively) (Fig. 5).

### Inflammation biomarkers

#### Parotid glands

**Fig. 6:**
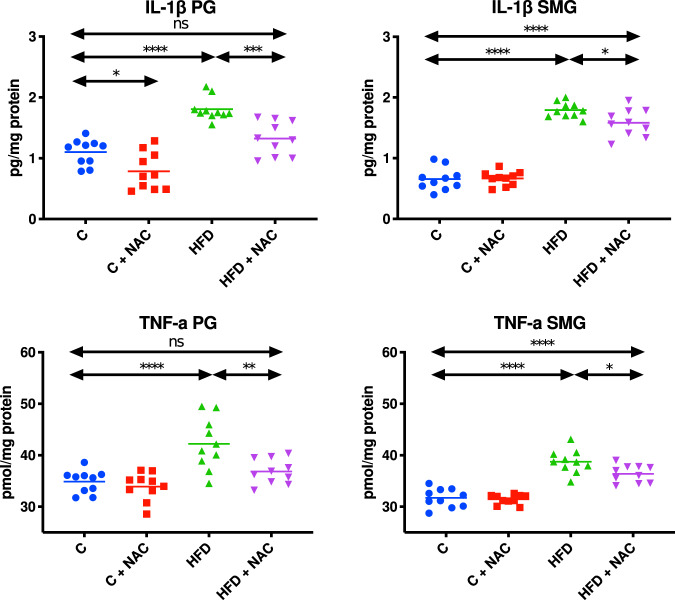
Impact of N-acetylcysteine (NAC) administration on inflammation biomarkers in the salivary gland mitochondria. *p < 0.05, **p < 0.005, ***p < 0.0005, ****p < 0.0001. C control rats, C + NAC control rats + NAC administration, HFD rats fed the high-fat diet, HFD + NAC rats fed the high-fat diet + NAC administration, IL-1β interleukin 1β, ns not significant, PG parotid salivary glands, SMG submandibular salivary glands, TNF-α tumor necrosis factor α.

The concentrations of both IL-1β and TNF-α in the mitochondrial fraction of the parotid salivary glands of HFD rats were considerably enhanced compared to the control group (↑39% *p* < 0.0001, ↑17% *p* < 0.0001, respectively). NAC administration effectively prevented the increase of mitochondrial IL-1β and TNF-α content as the concentrations of both these cytokines in the parotid glands of HFD + NAC rats did not differ significantly compared to the control group but were considerably lower than IL-1β and TNF-α concentrations in the mitochondrial fraction of the parotid glands of HFD rats (↓27% *p* = 0.0007, ↓13% *p* = 0.0042, respectively) (Fig. 6).

#### Submandibular glands

Both IL-1β and TNF-α concentrations in the mitochondrial fraction of the submandibular salivary glands of HFD rats were considerably enhanced compared to the control group (↑64% *p* < 0.0001, ↑18% *p* < 0.0001, respectively). NAC administration did not effectively prevent the increase of mitochondrial IL-1β and TNF-α levels as the concentrations of both these cytokines in the submandibular glands of HFD + NAC rats were markedly higher compared to the control group (↑59% *p* < 0.0001, ↑13% *p* < 0.0001, respectively). However, it should be noted that they were significantly lower compared to IL-1β and TNF-α concentrations in the mitochondrial fraction of the submandibular glands of HFD rats (↓12% *p* = 0.0404, ↓6% *p* = 0.0239, respectively) (Fig. 6).

### Apoptosis parameters

#### Parotid glands

**Fig. 7:**
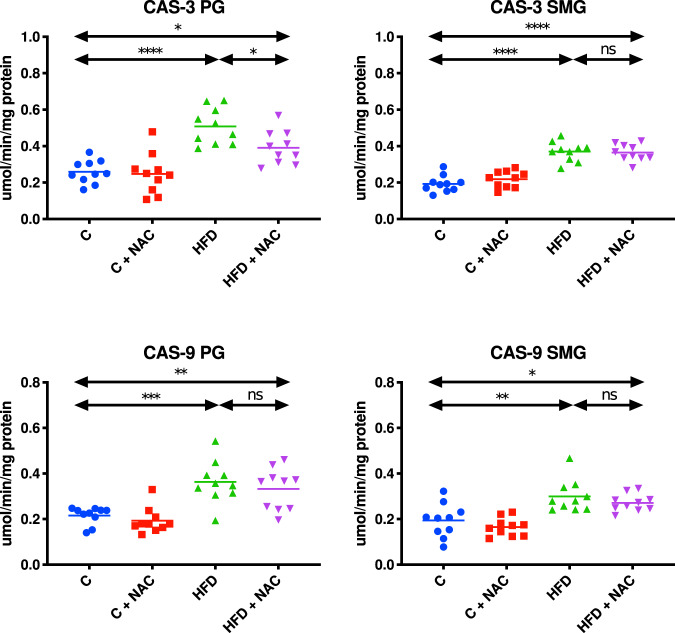
Impact of N-acetylcysteine (NAC) administration on the activity of caspases in the salivary gland mitochondria. *p < 0.05, **p < 0.005, ***p < 0.0005, ****p < 0.0001. C control rats, C + NAC control rats + NAC administration, CAS-3 caspase 3, CAS-9 caspase 9, HFD rats fed the high-fat diet, HFD + NAC rats fed the high-fat diet + NAC administration, ns not significant, PG parotid salivary glands, SMG submandibular salivary glands.

The activities of both CAS-3 and CAS-9 in the mitochondrial fraction of the parotid salivary glands of HFD rats were significantly higher compared to the control group (↑49 % *p* < 0.0001, ↑41% *p* = 0.0004, respectively). CAS-3 activity in the mitochondrial fraction of the parotid glands of HFD + NAC rats was considerably lower compared to the HFD group (↓23% *p* = 0.0375), and it was not different compared to the control group. AC administration did not prevent an increase in mitochondrial CAS-9 concentration, which led to the significantly higher activity of this enzyme in the parotid glands of the HFD + NAC group compared to the control rats (↑35% *p* = 0.0055). CAS-3 activity did not differ from the HFD group (Fig. 7).

**Fig. 8:**
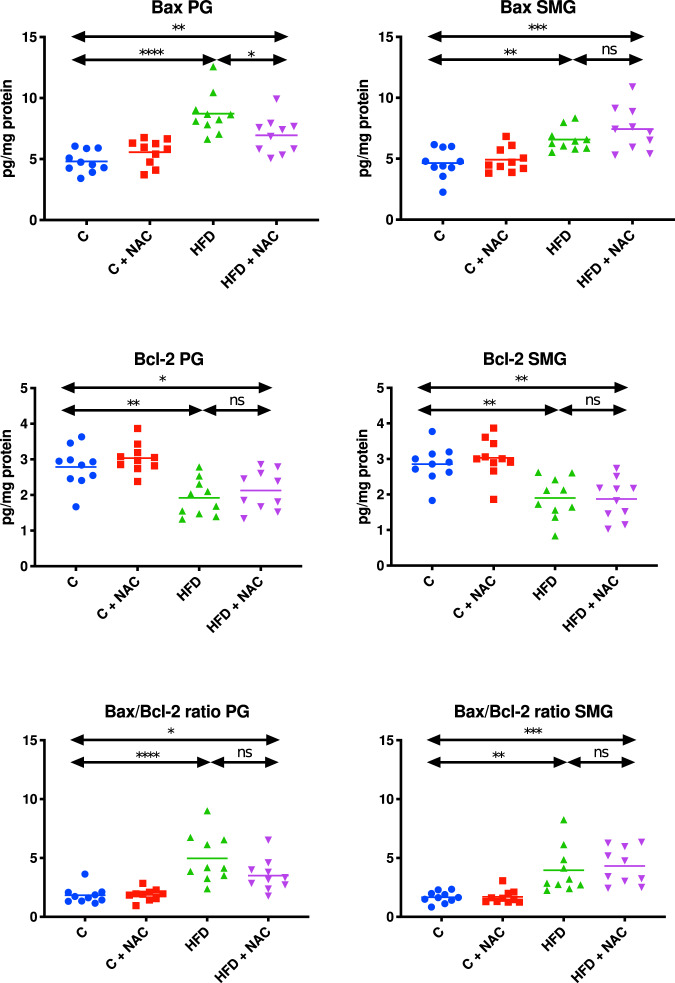
Impact of N-acetylcysteine (NAC) administration on apoptotic proteins in the salivary gland mitochondria. *p < 0.05, **p < 0.005, ***p < 0.0005, ****p < 0.0001. Bax pro-apoptotic protein, Bcl-2 anti-apoptotic protein, C control rats, C + NAC control rats + NAC administration, HFD rats fed the high-fat diet, HFD + NAC rats fed the high-fat diet + NAC administration, ns not significant, PG parotid salivary glands, SMG submandibular salivary glands.

The concentration of Bax and Bcl-2, as well as the Bax/Bcl-2 ratio in the mitochondrial fraction of the parotid salivary glands of HFD rats, was significantly higher compared to the control group (↑45% *p* < 0.0001, ↑31% *p* = 0.0029, ↑62% *p* < 0.0001, respectively). NAC administration added at the stage of developed hyperglycemia did not counteract an increase in Bax and Bcl-2 levels as well as the Bax/Bcl-2 ratio in the mitochondrial fraction of the parotid glands of rats, which resulted in considerably higher concentrations of Bax, Bcl-2, and the Bax/Bcl-2 ratio compared to the controls (↑31% *p* = 0.0052, ↑24% *p* = 0.0315, ↑48% *p* = 0.0310, respectively). Only Bax concentration in the mitochondrial fraction of the parotid glands of rodents from the HFD + NAC group was significantly lower compared to the HFD group (↓20% *p* = 0.0244). Mitochondrial Bcl-2 concentration and the Bax/Bcl-2 ratio did not differ between HFD + NAC and HFD groups (Fig. 8).

#### Submandibular glands

Both CAS-3 and CAS-9 activities in the mitochondrial fraction of the submandibular salivary glands of HFD rats were considerably enhanced compared to the control group (↑48% *p* < 0.0001, ↑35% *p* = 0.0012, respectively). NAC did not prevent the increase in mitochondrial CAS-3 and CAS-9 concentrations, which resulted in a markedly higher activity of these enzymes in the submandibular glands of HFD + NAC rats compared to the control group (↑47% *p* < 0.0001, ↑28% *p* = 0.0249, respectively). Mitochondrial CAS-3 and CAS-9 activities did not differ from the HFD group (Fig. 7).

The concentrations of Bax and Bcl-2, as well as the Bax/Bcl-2 ratio in the mitochondrial fraction of the submandibular salivary glands of HFD rats, was significantly higher compared to the control group (↑29% *p* = 0.0092, ↑33% *p* = 0.0024, ↑58% *p* = 0.002, respectively). NAC administration included at the stage of developed hyperglycemia did not prevent an increase of Bax and Bcl-2 concentrations or the Bax/Bcl-2 ratio in the mitochondrial fraction of the submandibular glands of rats, as a result of which Bax and Bcl-2 levels and the Bax/Bcl-2 ratio were considerably enhanced compared to the control group (↑37% *p* = 0.0001, ↑34% *p* = 0.0017, ↑61 % *p* = 0.0003, respectively). However, they did not differ from their values obtained in the HFD group (Fig. 8).

### Inflammation and apoptosis mRNA expression

#### Parotid glands

**Fig. 9:**
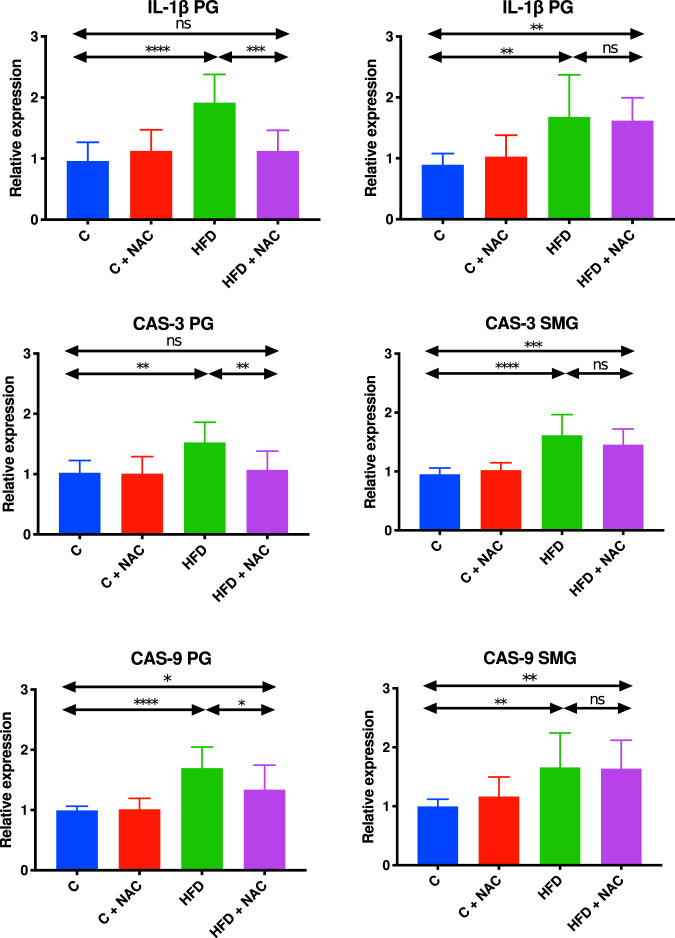
Impact of N-acetylcysteine (NAC) administration on the mRNA expression of inflammation and apoptosis biomarkers in the whole salivary glands. *p < 0.05, **p < 0.005, ***p < 0.0005, ****p < 0.0001. C control rats, C + NAC control rats + NAC administration, CAS-3: caspase 3 CAS-9 caspase 9, HFD rats fed the high-fat diet, HFD + NAC rats fed the high-fat diet + NAC administration, IL-1β interleukin 1β, ns not significant, PG parotid salivary glands, SMG submandibular salivary glands.

mRNA expression of IL-1 β, CAS-3, and CAS-9 were significantly higher in the parotid glands of hyperglycemic rats compared to controls (↑50% *p* < 0.0001, ↑33% *p* = 0,002, ↑42% *p* < 0.0001, respectively). NAC administration in the HFD group led to a significant decrease in IL-1 β and CAS-3 expression to the levels observed in healthy rats (↓41% *p* = 0.0002, ↓30% *p* = 0.0056, respectively). Despite the fact that in the HFD + NAC group there was a significant decrease in the CAS-9 expression compared to the HFD group (↓21% *p* = 0.0379, respectively), its level was still significantly higher compared to the control group (↑26% *p* = 0.0492, respectively) (Fig. 9).

#### Submandibular glands

mRNA expression of IL-1 β, CAS-3, and CAS-9 was significantly higher in the submandibular glands of HFD rats compared to the control group (↑47% *p* = 0.0017, ↑41% *p* < 0.0001, ↑40% *p* = 0.0058, respectively). NAC administration in rats with hyperglycemia did not lead to a significant reduction in the expression of the above-mentioned parameters. The level of IL-1 β, CAS-3, and CAS-9 mRNA expression was still significantly higher compared to the control group (↑45% *p* = 0.0042, ↑35% *p* = 0.0002, ↑39% *p* = 0.0078, respectively) (Fig. 9).

### Glutathione

#### Parotid glands

**Fig. 10:**
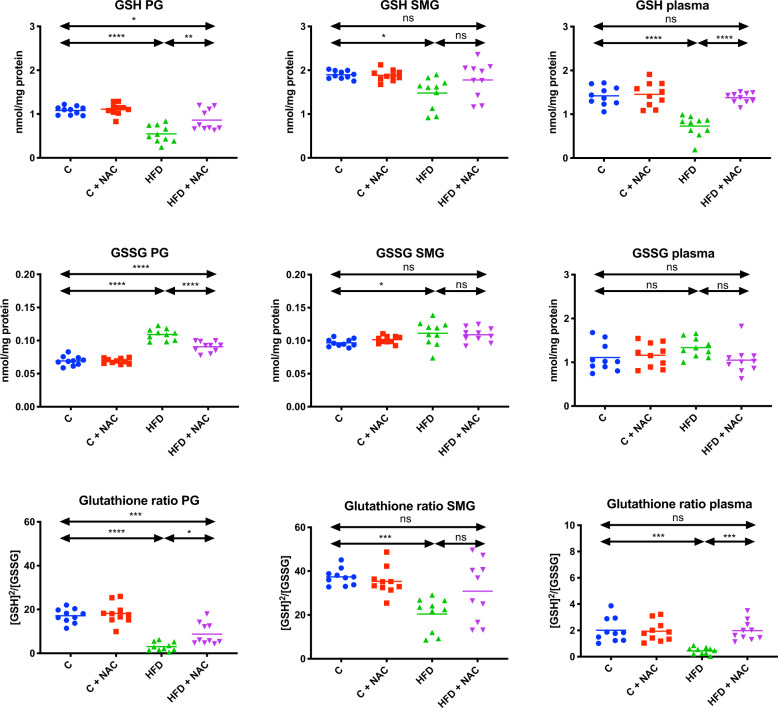
Impact of N-acetylcysteine (NAC) administration on glutathione metabolism in the salivary gland mitochondria and plasma. *p < 0.05, **p < 0.005, ***p < 0.0005, ****p < 0.0001. C control rats, C + NAC control rats + NAC administration, HFD rats fed the high-fat diet, HFD + NAC rats fed the high-fat diet + NAC administration, GSH reduced glutathione, GSSG oxidized glutathione, ns not significant, PG parotid salivary glands, SMG submandibular salivary glands.

In the mitochondrial fraction of the parotid salivary glands, GSH concentration and the glutathione ratio were significantly lower (↓49 % *p* < 0.0001, ↓82% *p* < 0.0001, respectively), and the GSSG level were significantly higher (↑36% *p* < 0.0001) in HFD rats compared to the control. NAC administration of hyperglycemic rats did not prevent adverse changes in mitochondrial reduced glutathione concentration and the GSH/GSSG ratio, as GSH and the glutathione ratio were still considerably lower than in the control group (↓21% *p* = 0.0348, ↓49% *p* = 0.0002, respectively). It is noteworthy, however, that these values were significantly higher than in the HFD group (↑36% *p* = 0.0016, ↑65% *p* = 0.0123, respectively). NAC administration did not reduce mitochondrial GSSG pool to the levels observed in the control group, as a result of which GSSG concentration in the HFD + NAC group was markedly higher than in the control group (↑24% *p* < 0.0001), but significantly lower than in the HFD group (↓17% *p* < 0.0001) (Fig. 10).

#### Submandibular glands

GSH concentration and the glutathione ratio were significantly lower (↓22% *p* = 0.0102, ↓45% *p* = 0.0006, respectively), and GSSG was considerably higher (↑13% *p* = 0.0368) in the mitochondrial fraction of the submandibular salivary glands of HFD rats compared to the controls. NAC administration of hyperglycemic rats prevented unfavorable changes in mitochondrial reduced glutathione level, the glutathione ratio, and GSSG concentration, as GSH and GSSG concentrations, as well as the glutathione ratio, did not differ significantly from similar assays performed in the control group (Fig. 10).

#### Plasma

GSH concentration and glutathione ratio were significantly lower (↓49% *p* < 0.0001, ↓78% *p* < 0.0001, respectively) in the plasma of HFD rats compared to the control. NAC administration of rats with HFD prevented unfavorable changes in the concentration of reduced glutathione and glutathione ratio, reaching values similar to the control group (↓47% *p* < 0.0001, ↓77% *p* = 0.0002, respectively). We showed no significant difference in GSSG concentration between the HFD group and the control group (Fig. 10).

### Enzymatic antioxidants and oxidative stress

#### Parotid glands

**Fig. 11:**
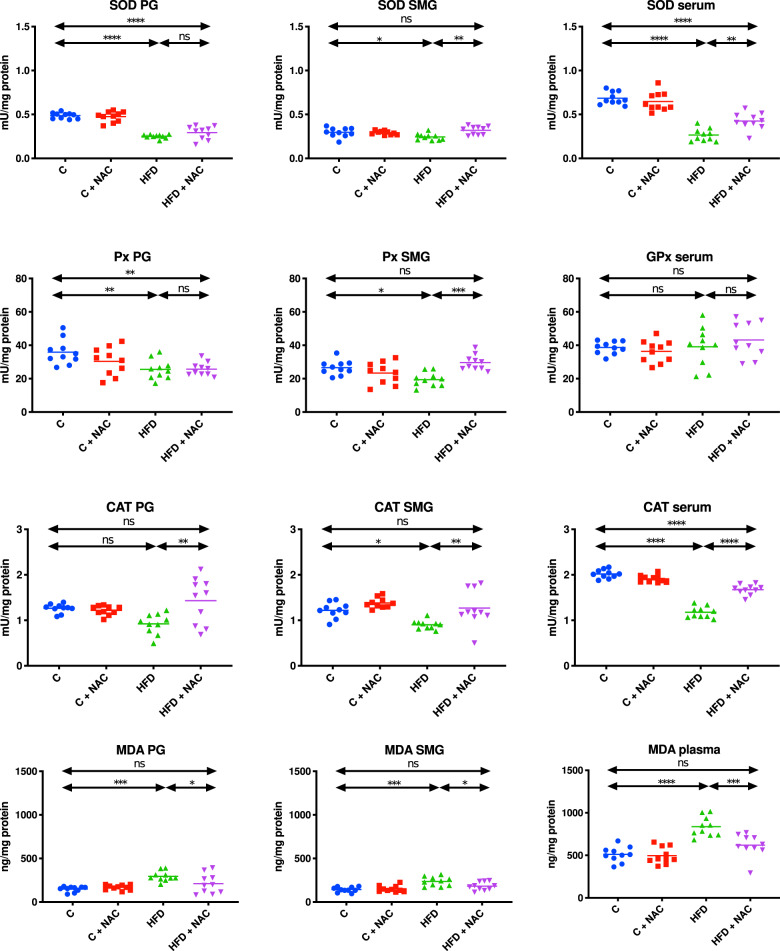
Impact of N-acetylcysteine (NAC) administration on enzymatic antioxidants and oxidative stress in the salivary gland mitochondria and plasma. *p < 0.05, **p < 0.005, ***p < 0.0005, ****p < 0.0001. C control rats, C + NAC control rats + NAC administration, HFD rats fed the high-fat diet, HFD + NAC rats fed the high-fat diet + NAC administration, ns not significant, PG parotid salivary glands, SMG submandibular salivary glands, SOD superoxide dismutase, Px peroxidase, GPx glutathione peroxidase, CAT catalase, MDA malondialdehyde.

The activity of SOD and Px was significantly lower and the concentration of MDA was significantly higher in the mitochondrial fraction of the parotid glands of HFD rats compared to the control (↑48% *p* < 0.0001, ↑29% *p* = 0.007, ↑50% *p* = 0.0001, respectively). NAC administration of hyperglycemic rats did not prevent changes in reduced mitochondrial SOD and Px activity, since SOD and Px were still significantly lower than in the control group (↓40% *p* < 0.0001, ↓28% *p* = 0.008, respectively). Nevertheless, the HFC + NAC group had a significantly lower concentration of mitochondrial MDA compared to the HFD group, reaching values similar to the control group (↓28% *p* = 0.0373, respectively). There were no significant differences in CAT activity in the mitochondrial fraction of the parotid glands of HFD rats compared to the control, but administration with NAC caused a significant increase in CAT activity in the HFD + NAC group compared to the HFD group (↑36% *p* = 0.0017, respectively) (Fig. 11).

#### Submandibular glands

The mitochondrial fraction of the submandibular glands of HFD rats was characterized by significantly lower SOD, Px, and CAT activity and significantly higher MDA concentration compared to the control group (↓18% *p* = 0.0391, ↓27% *p* = 0.0136, ↓26% *p* = 0.0197, ↑39% *p* = 0.0001, respectively). NAC administration significantly increased the activity of mitochondrial SOD, Px, and CAT and lowered the concentration of MDA to the levels observed in the control group (↑23% *p* = 0.0016, ↑34% *p* = 0.0003, ↑29% *p* = 0.0055, ↓22% *p* = 0.0405, respectively) (Fig. 11).

#### Serum/plasma

Although there was no significant difference in the activity of serum GPx, the activity of serum SOD and CAT in the group of insulin-resistant rats was significantly lower compared to the control group (↓61% *p* < 0.0001, ↓42% *p* < 0.0001, respectively). NAC administration significantly increased the activity of SOD and CAT compared to the HFD group but did not reach levels similar to the control group (↑38% *p* = 0.0012, ↑29% *p* < 0.0001, respectively). The concentration of MDA in the group of rats fed a high-fat diet was significantly higher compared to the control group (↑39% *p* < 0.0001, respectively). Administration with NAC led to a significant reduction in the concentration of MDA in the plasma of HFD rats reaching levels similar to the control group (↓26% *p* = 0.0006, respectively) (Fig. 11).

### Histological studies

**Fig. 12:**
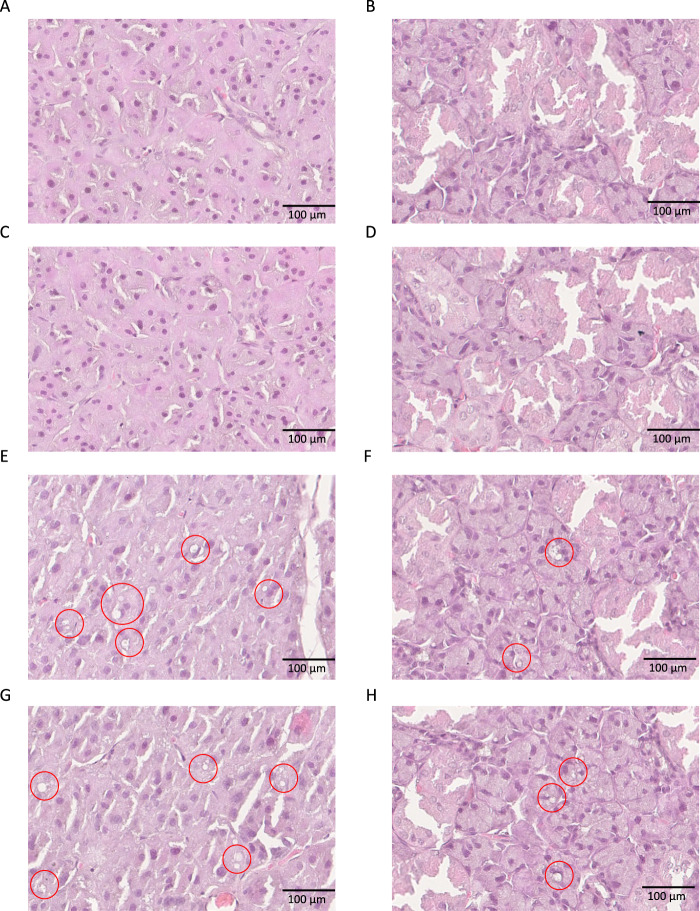
Impact of N-acetylcysteine (NAC) administration on histological observation of rat salivary glands. **A** Parotid salivary glands of control rats; **B** submandibular salivary glands of control rats; **C** parotid salivary glands of C + NAC rats; **D** submandibular salivary glands of C + NAC rats; **E** parotid salivary glands of HFD rats; **F** submandibular salivary glands of HFD rats; **G** parotid salivary glands of HFD + NAC rats; **H** submandibular salivary glands of HFD + NAC rats. Red circles indicate vacuoles. C control rats, C + NAC control rats + NAC administration, HFD rats fed the high-fat diet, HFD + NAC rats fed the high-fat diet + NAC administration.

In general, the histological structure of salivary glands did not differ between groups. However, both parotid and submandibular glands of HFD rats showed degenerative changes in the form of vacuolation. They are more marked in the parotid gland than in the submandibular gland. Importantly, NAC administration did not reduce these alterations (Fig. 12, Table 2).

**Table 2:** Effect of NAC supplementation on histological observation of rat salivary glands.

	C	C + NAC	HFD	HFD + NAC
Parotid glands	10 (+)	10 (+)	6 (++++), 4 (+++++)*	5 (++++), 5 (+++++)*
Submandibular glands	10 (+)	10 (+)	7 (++), 3 (+++)*	8 (++), 2 (+++)*

C control rats C + NAC control rats + N-acetylcysteine, HFD rats fed the high-fat diet, HFD + NAC rats fed the high-fat diet + N-acetylcysteine, + single vacuoles in the cytoplasm of salivary acinar cells, ++ 5–10% of each section occupied by pathological alternations, +++ 11–20% of each section occupied by pathological alternations, ++++ 21–30% of each section occupied by pathological alternations, +++++ >30% of each section occupied by pathological alternations.

### Correlations

In the HFD + NAC group we showed a negative correlation between the ADP/ATP ratio in parotid gland mitochondria and stimulated saliva secretion (*r* = −0.886, *p* = 0.0006), as well as a positive correlation between the activity of complex II + III in the mitochondria of this salivary gland and stimulated saliva secretion (*r* = 0.819, *p* = 0.0038). In addition, in the parotid glands of HFD + NAC rats we observed a negative correlation between mitochondrial CAS-9 activity and GSH concentration (*r* = −0.649, *p* = 0.042), and a positive correlation between GSH concentration and the activity of complex II + III (*r* = 0.775, *p* = 0.0084) as well as between TNF-α concentration and the Bax/Bcl-2 ratio (*r* = 0.853, *p* = 0.002).

In the HFD + NAC group, a positive correlation was found between COX activity and the ADP/ATP ratio in the submandibular gland mitochondria (*r* = 0.719, *p* = 0.0192)

Moreover, in both salivary glands there was a negative correlation between the activity of mitochondrial complex II + III and DCFH-DA concentration (PG: *r* = −0.786, *p* = 0.007; SMG: *r* = −0.509 *p* = 0.05), and a positive correlation between DCFH-DA concentration and caspase 9 activity (PG: *r* = 0.621, *p* = 0.05; SMG: *r* = 0.901, *p* < 0.0001).

## Discussion

Previous studies demonstrated that rats fed the high-fat diet for 6 weeks developed hyperglycemia [2, 3, 33]. The inclusion of a 4-week NAC supplementation at this stage, despite the continuation of the HFD, prevented the development of IR and strengthened the cytoplasmic antioxidant systems of salivary glands [33]. Interestingly, NAC blocked oxidative modifications of proteins and carbohydrates and did not reduce lipid peroxidation in the cytoplasm of acinar cells of the submandibular glands of rats fed the HFD vs. the control rats. In the cytoplasm of acinar cells of the parotid glands, NAC supplementation reduced the intensity of lipid peroxidation compared to the HFD group, but these values were significantly higher than those observed in the control group [33]. NAC supplementation did not entail the reduction of carbohydrate and protein oxidation in the cytoplasm of acinar cells of the parotid glands. In conclusion, NAC-dependent reduction of cytoplasmic OS was only partial and dependent on the type of the salivary gland [33]. The persistence of OS despite NAC administration is probably a result of excessive ROS formation, but this hypothesis needs to be examined and explained. The main salivary source of ROS/RNS are processes generated in the mitochondria during cellular respiration as well as oxidative reactions catalyzed by oxidative enzymes [34]. A significant ROS source in the saliva is inflammatory mediators: NOX, IL-1β, TNF-α, IL-6, suPAR, or galectin-3 [34–38]. The intracellular ROS/RNS generation is also associated with mitochondrial apoptosis [2, 8]. However, the redox homeostasis of salivary gland cells may differ from the mitochondrial oxidoreductive balance. Therefore, it is essential to evaluate energy production, antioxidant barrier, oxidative damage, inflammation, and apoptosis not in the salivary gland homogenate but in the mitochondria themselves.

The results presented herein are innovative and allow us to assess the activity of the main sources of ROS/RNS: respiratory chain enzymes, NOX, and XO as well as the processes accompanied by ROS formation, i.e., inflammation and apoptosis in the salivary glands of rats in whom NAC supplementation was introduced at the stage of HFD-induced hyperglycemia. We also tried to determine if NAC supplementation could reverse the harmful effect of HFD on mitochondrial function, apoptosis, and inflammation in the salivary glands of hyperglycaemic rats. Our previous paper showed that the inclusion of NAC supplementation at the stage of hyperglycemia prevented the dysfunction of the salivary glands only to a limited extent [33]. However, this study was performed only in salivary gland homogenates and the cause of the decreased function of the salivary glands is still unknown. Therefore, the current study was an attempt to assess whether salivary gland dysfunction in HFD + NAC rats can be caused by mitochondrial dysfunction.

Generally, the results obtained in the group fed the HFD are consistent with our previous observations [2]. We were surprised by the fact that HFD-induced alteration in the functioning of respiratory chain complexes was not normalized after the introduction of NAC supplementation at the stage of hyperglycemia. One of the most important processes that occur in the mitochondria is oxidative phosphorylation, during which electrons are removed from energy substrates and transported to oxygen. The process is accompanied by ATP generation by the system of mitochondrial complexes (I–IV). Approximately, 0.1–1% of the oxygen consumed in the mitochondria is transformed to superoxide anion, which is then converted to hydrogen peroxide and water. Under physiological conditions, complexes I and III are responsible for oxygen reduction to superoxide anion [39]. According to our results, NAC supplementation applied at the stage of induced hyperglycemia recreated the mitochondrial chain function in both salivary glands of HFD + NAC rats to a small extent. Only the activity of complex I and COX in the parotid glands of these rats was completely normalized by NAC. The observed partial recovery of complex II + III function in the parotid gland mitochondria of NAC-treated rats could reflect the decreased GSH concentration, which seems to be confirmed by a positive correlation between GSH concentration and the activity of this complex. In the submandibular glands of HFD + NAC rats, NAC supplementation did not rescue the activity of complexes I and II + III and COX at all. Their values, exception COX, remained at the level similar to the activity of these complexes in the group of HFD rats, and lower—compared to the control group. As mitochondrial COX is of transcendental importance with respect to energy metabolism [40], a positive correlation between the activity of COX and the ADP/ATP ratio and HFD-induced increase in COX activity in the submandibular glands might contribute to the impairment of ATP production in this salivary gland. It should be highlighted that increased ADP/ATP ratio in the salivary glands of HFD + NAC group can result from sustained reduced activity of CS—an enzyme associated with the cycle of tricarboxylic acid which, under physiological conditions, contributes to the delivery of 12 ATP molecules. Negative correlations between the activity of complex II + III and DCFH-DA concentration in both salivary glands suggest, on the one hand, that reduced activity of this complex entails the increased generation of mitochondrial ROS. This, in turn, can maintain OS despite NAC supplementation. On the other hand, they may prove that excessive ROS production is crucial for the induction of mitochondrial alteration.

It is possible that HFD decreases enzymatic activities of respiratory chain subunits, owing to decreased number of fully constructed complexes as a result of the reduced rate of their synthesis, as observed in the liver of mice with induced non-alcoholic steatohepatitis [1]. In the case of salivary glands, these changes may be irreversible, or the lack of evidence of any particular effect of NAC on the mitochondrial system cannot reflect the in vivo condition. It was demonstrated that precise functioning of mitochondria, especially NAD^+^-dependent dehydrogenases, as well as transport of the reducing equivalent through NADH are lost during operation in isolated mitochondria [41].

It is assumed that intracellular redox homeostasis is largely dependent on enzymatic antioxidant activity and GSH concentration. Moreover, the GSH/GSSG ratio represents the main redox buffer and is, therefore, an indicator of redox balance in a cell. According to Luschack [42], elevated OS severity is manifested by increased concentration of GSSG as well as decreased content of GSH and GSH/GSSG ratio. Direct GSH supplementation is not recommended because GSH is not transported inside the cells—it is synthesized in them [43]. Although it has been evidenced that one of the best sources of disulfide groups for GSH regeneration is NAC [44]. In our experiment, NAC supplementation restored GSH/GSSG balance solely in the mitochondria of the submandibular salivary glands and in the plasma. Reduced activities of mitochondrial antioxidant enzymes (↓SOD, ↓Px, ↓CAT) in the HFD group are normalized only in the submandibular salivary glands of rats supplemented with NAC. In the parotid gland, only the mitochondrial CAT, due to NAC supplementation, equals the enzyme activity in control salivary glands. In the parotid gland mitochondria, despite an undoubtedly significant improvement of these parameters compared to the HFD group (↑59% GSH, ↓19% GSSG, ↑186% GSH/GSSG ratio), we still observed considerably reduced GSH concentration (↓21%), increased GSSG (↑31%) and decreased GSH/GSSG ratio (↓50%) compared to the control group. The reason for the obtained differences between the examined salivary glands remains unknown, and the results of our study do not explain this phenomenon. GSH is synthesized solely in the cytoplasm and can easily permeate through the outer mitochondrial membrane through the porin channels, but as an anion, it cannot diffuse through the inner mitochondrial membrane. At least two systems transporting GSH into mitochondria through the inner membrane are known [45]. Their efficient functioning ensures a suitable concentration of GSH in the mitochondria. Interestingly, histological studies of Kołodziej et al. [46]. demonstrated increased fatty vacuolization of the parotid gland cells and minimal morphological changes in the submandibular glands of rats with hyperglycemia induced by the HFD. Our study confirms these observations. Ittichaicharoen et al. [47]. observed that a 16-week HFD results in mitochondrial edema, reduced number of cristae, and depolarization of the inner mitochondrial membrane of salivary glands. Perhaps HFD/hyperglycemia-induced morphological disturbances of the mitochondria in the parotid glands, as opposed to submandibular glands, are irreversible and persist despite NAC supplementation. On the other hand, there is a probability of oxidative damage to the enzymes responsible for GSH synthesis and too slow de novo synthesis, selectively in the parotid glands. Interestingly, it has been observed that GSH deficiency is associated with the activation of caspases [48] and stimulation of the apoptotic pathway, which is confirmed by the negative correlation between CAS-9 activity and GSH concentration in the parotid glands of HFD + NAC rats. The positive correlation between DCFH-DA and caspase 9 activity and increased cascade 3 and 9 activity in both salivary glands (vs. the control group) suggests that the apoptotic pathway, most likely due to increased ROS formation, is not inhibited by NAC supplementation. However, it should be emphasized that RT-PCR showed that the relative expression of both CAS-3 and -9 in the mitochondria of the parotid gland is reduced after NAC supplementation compared to the group exposed to HFD. In our study apoptosis parameters in the salivary glands of rats supplemented with NAC were virtually unchanged compared to the group fed only the HFD, without NAC supplementation. These parameters remained significantly higher compared to the control group (in the parotid glands: ↑44% Bax, ↓24% Bcl-2, ↑91% Bax/Bcl-2 ratio; in the submandibular glands: ↑57% Bax, ↓35% Bcl-2, ↑157% Bax/Bcl-2 ratio). These results are consistent with the observations suggesting that the statement that NAC is solely an anti-apoptotic agent is probably an over-generalization [49]. The intensity of apoptosis processes is more pronounced in the mitochondria of the parotid glands compared to the submandibular glands of HFD + NAC rats. Perhaps the stimulation of apoptotic pathways results from the inability of both salivary glands of HFD + NAC rats to maintain adequate ATP concentration (the above-mentioned significant decrease of the ADP/ATP ratio vs. the control in the parotid as well as submandibular salivary glands). Bailey et al. [50] showed that reduced ATP concentration leads to intensified hepatocyte apoptosis. The positive correlation between TNF-α concentration and the Bax/Bcl-2 ratio in the parotid glands of HFD + NAC rats suggests that lower intensity of apoptosis may be caused by the fact that parotid gland inflammation in these rats is suppressed compared to the control group (NOX activity, IL-1β, and TNF-α concentrations comparable to the controls). In the submandibular salivary glands, despite a significant decrease in NOX activity and IL-1β and TNF-α concentrations as compared to the HFD group, these values were considerably higher than in the control rats. The elimination/reduction of an inflammatory state is most likely an effect of NAC-dependent inhibition of NF-κB activity. NF- κB enhances the production of proinflammatory cytokines [51]. Furthermore, NF-κB regulates NOX activity by inducing the expression of the phox subunit for phagocytic oxidase gp91phox [52].

NAC supplementation reduced the lipid peroxidation process (MDA) in the mitochondria of both salivary glands and in the plasma of hyperglycemic rats to the levels observed in the control group. This result may indicate reduced ROS/RNS generation. It should be remembered that the molecules of membrane lipids undergo peroxidation at higher concentrations of ROS than proteins [53]. It cannot be also assumed that the elimination of OS is complete, a single marker of oxidative damage has a limited diagnostic and prognostic value.

Salivary biomarkers are often used in the diagnosis of oral and general diseases [35–37, 54–59]. In the present study, we have shown no correlation between the markers of OS (antioxidants and MDA concentration) in the plasma and the mitochondria of the salivary glands. According to us, the demonstrated lack of dependence suggests that changes in the activity/concentration of redox equilibrium components are the result of pathological processes in the mitochondria of the salivary glands, regardless of systemic changes.

We observed the negative correlation between the ADP/ATP ratio in the mitochondria of the parotid gland and SWS as well as the positive correlation between the activity of complex II + III in the mitochondria of this salivary gland and SWS. These correlations may suggest that SWS is closely related to the mitochondrial respiratory capacity and appropriate ATP level, which is consistent with the observations of Xiang et al. [60]. What is more, Ittichaicharoen et al. [47] proved that mitochondria play a key role in the regulation of the Ca^2+^ mobilization pathway in human follicular cells of salivary glands, which is a prerequisite for saliva secretion.

**Fig. 13:**
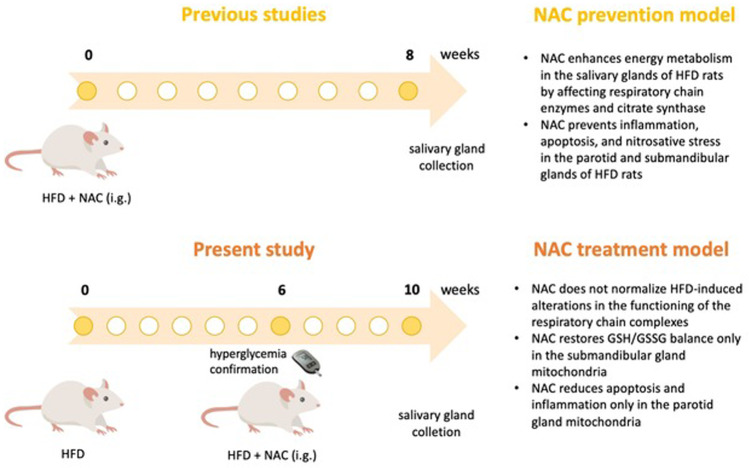
Graphical conclusions from present and previous studies regarding N-acetylcysteine (NAC) action on salivary glands.

Our results indicate that in a rat model of experimental IR, NAC administration after the induction of a hyperglycemic state provided little protection of the salivary gland mitochondria against free radical injury, apoptosis, and inflammation. We did not observe the expected effects, which was rather not due to the lack of NAC action in the salivary gland mitochondria of rats. It seems that in the salivary glands the effects of NAC supplementation depend on the timing of the start of its administration, i.e., the earlier the start of NAC supplementation, the more satisfactory effects. We demonstrated, as mentioned in the introduction, that the inclusion of NAC supplementation at the same time as exposing rats to the HFD resulted in respiratory chain normalization, i.e., it restored the activity of the complexes, reduced the ADP/ATP ratio, decreased ROS production, weakened apoptotic pathways, rescued GSH pool and prevented cytokine production to the levels observed in the control salivary glands [8] (Fig. 13).

## Conclusions

1. NAC supplementation does not rescue HFD-induced alteration of the respiratory chain complexes in the salivary glands of hyperglycaemic rats.
2. The reduced activity of mitochondrial complex II + III entails the increased generation of mitochondrial ROS despite NAC supplementation. This persistent alteration may maintain OS in the salivary glands.
3. NAC supplementation in the current experiment scheme restores GSH/GSSG balance and activity of antioxidants enzymes only in the mitochondria of the submandibular salivary glands.
4. NAC supplementation in the current experiment scheme reduces the lipid peroxidation process in the mitochondria of both salivary glands and the plasma of HFD rats to the levels of the control group.
5. Introducing NAC supplementation at the stage of HFD-induced hyperglycemia reduces apoptosis and inflammation only in the mitochondria of the parotid glands.
6. SWS in rats from the HFD + NAC group is closely related to mitochondrial respiratory capacity and appropriate ATP level.

## Data Availability

The article contains complete data used to support the findings of this study.

## References

[1] García-Ruiz I, Solís-Muñoz P, Fernández-Moreira D, Grau M, Colina F, Muñoz-Yagüe T (2014). High-fat diet decreases activity of the oxidative phosphorylation complexes and causes nonalcoholic steatohepatitis in mice. Dis Model Mech.

[2] Zalewska A, Ziembicka D, Zendzian-Piotrowska M, Maciejczyk M. The impact of high-fat diet on mitochondrial function, free radical production, and nitrosative stress in the salivary glands of wistar rats. Oxid Med Cell Longev. 2019; 10.1155/2019/2606120 10.1155/2019/2606120 PMC6637679 31354904

[3] Zalewska A, Knaś M, Zendzian-Piotrowska M, Waszkiewicz N, Szulimowska J, Prokopiuk S (2014). Antioxidant profile of salivary glands in high fat diet-induced insulin resistance rats. Oral Dis.

[4] Anupam K, Kaushal J, Prabhakar N, Bhatnagar A (2018). Effect of redox status of peripheral blood on immune signature of circulating regulatory and cytotoxic T cells in streptozotocin induced rodent model of type I diabetes. Immunobiology.

[5] Brownlee M (2001). Biochemistry and molecular cell biology of diabetic complication. Nature.

[6] Bayrami G, Alihemmati A, Karimi P, Javadi A, Keyhanmanesh R, Mohammadi M (2018). Combination of vildagliptin and ischemic postconditioning in diabetic hearts as a working strategy to reduce myocardial reperfusion injury by restoring mitochondrial function and autophagic activity. Adv Pharm Bull.

[7] Bullon P, Newman HN, Battino M (2014). Obesity, diabetes mellitus, atherosclerosis and chronic periodontitis: a shared pathology via oxidative stress and mitochondrial dysfunction. Periodontology.

[8] Zalewska A, Szarmach I, Zendzian-Piotrowska M, Maciejczyk M. The effect of N-acetylcysteine on respiratory enzymes, ADP/ATP ratio, glutathione metabolism, and nitrosative stress in the salivary gland mitochondria of insulin resistant rats. Nutrients. 2020;12, 10.3390/nu12020458 10.3390/nu12020458 PMC7071150 32059375

[9] Miquel J, Ferrandiz ML, De Juan E, Sevila I, Martinez M (1995). N-acetylcysteine protects against age-related decline of oxidative phosphorylation in liver mitochondria. Eur J Pharm.

[10] Cocco T, Sgobbo P, Clemente M, Lopriore B, Grattagliano I, Di Paola M (2005). Tissue-specific changes of mitochondrial functions in aged rats: effect of a long-term dietary treatment with N-acetylcysteine. Free Radic Biol Med.

[11] Xiong Y, Peterson PL, Lee CP (1999). Effect of N-acetylcysteine on mitochondrial function following traumatic brain injury in rats. J Neurotrauma.

[12] Żukowski P, M M, Matczuk J, Kurek K, Waszkiel D, Żendzian-Piotrowska M, et al. Effect of N-acetylcysteine on antioxidant defense, oxidative modification, and salivary gland function in a rat model of insulin resistance. Oxid Med Cell Longev. 2018; 10.1155/2018/6581970 10.1155/2018/6581970 PMC5831706 29636851

[13] Jaccob AA (2015). Protective effect of N-acetylcysteine against ethanol-induced gastric ulcer: a pharmacological assessment in mice. J Intercult Ethnopharmacol.

[14] Bligh EG, Dyer WJ (1959). A rapid method of total lipid extraction and purification. Can J Biochem Physiol.

[15] Maciejczyk M, Zebrowska E, Zalewska A, Chabowski A. Redox balance, antioxidant defense, and oxidative damage in the hypothalamus and cerebral cortex of rats with high fat diet-induced insulin resistance. Oxid Med Cell Longev. 2018; 10.1155/2018/6940515 10.1155/2018/6940515 PMC6146783 30271528

[16] Zalewska A, Maciejczyk M, Szulimowska J, Imierska M, Błachnio-Zabielska A. High-fat diet affects ceramide content, disturbs mitochondrial redox balance, and induces apoptosis in the submandibular glands of mice. Biomolecules. 2019; 10.3390/biom9120877 10.3390/biom9120877 PMC6995631 31847462

[17] Janssen AJ, Trijbels FJ, Sengers RC, Smeitink JA, van den Heuvel LP, Wintjes LT (2007). Spectrophotometric assay for complex I of the respiratory chain in tissue samples and cultured fibroblasts. Clin Chem.

[18] Rustin P, et al. Biochemical and molecular investigations in respiratory chain deficiencies. Clin Chim Acta. 1994; 10.1016/0009-8981(94)90055-8 10.1016/0009-8981(94)90055-8 7955428

[19] Muller FL, Liu Y, Van Remmen H. Complex III releases superoxide to both sides of the inner mitochondrial membrane. J Biol Chem. 2004; 10.1074/jbc.M407715200 10.1074/jbc.M407715200 15317809

[20] Wharton DC, Tzagoloff A. Cytochrome oxidase from beef heart mitochondria. Methods Enzymol. 1967; 10.1016/0075-6879(67)10048-7

[21] Srere PA Citrate synthase. [EC 4.1.3.7 Citrate oxaloacetate-lyase (CoA-acetylating)]. 1969.

[22] Griendling KK, Minieri CA, Ollerenshaw JD, Alexander RW (1994). Angiotensin II stimulates NADH and NADPH oxidase activity in cultured vascular smooth muscle cells. Circ Res.

[23] Prajda N, Weber G (1975). Malignant transformation-linked imbalance: decreased xanthine oxidase activity in hepatomas. FEBS Lett.

[24] Bondy SC, Guo SX (1994). Effect of ethanol treatment on indices of cumulative oxidative stress. Eur J Pharm.

[25] Maciejczyk M, Matczuk J, Żendzian-Piotrowska M, Niklińska W, Fejfer K, Szarmach I, et al. Eight-week consumption of high-sucrose diet has a pro-oxidant effect and alters the function of the salivary glands of rats. Nutrients. 2018;10; 10.3390/nu10101530 10.3390/nu10101530 PMC6212933 30336621

[26] Meki AR, M. A, Emade El Dein F, Hussein EAA, Hassanein HM (2004). Caspase-3 and heat shock protein-70 in rat liver treated with aflatoxin B1: effect of melatonin. Toxicon.

[27] Griffith OW (1980). Determination of glutathione and glutathione disulfide using glutathione reductase and 2-vinylpyridine. Anal Biochem.

[28] Misra HP, Fridovich I (1972). The role of superoxide anion in the autoxidation of epinephrine and a simple assay for superoxide dismutase. J Biol Chem.

[29] Mansson- Rahemtulla B, Baldone DC, Pruitt KM, Rahemtulla F (1986). Specific assays for peroxidases in human saliva. Arch Oral Biol.

[30] Aebi H (1984). Catalase in vitro. Methods Enzymol.

[31] Buege JA, Aust SD (1978). Microsomal lipid peroxidation. Methods Enzymol.

[32] Maciejczyk M, Kossakowska A, Szulimowska J, Klimiuk A, Knaś M, Car H (2017). Lysosomal exoglycosidase profile and secretory function in the salivary glands of rats with streptozotocin-induced diabetes. J Diab Res.

[33] Zalewska A, Zięba S, Kostecka-Sochoń P, Kossakowska A, Żendzian-Piotrowska M, Matczuk J (2020). NAC supplementation of hyperglycemic rats prevents the development of insulin resistance and improves antioxidant status but only alleviates general and salivary gland oxidative stress. Oxid Med Cell Longev.

[34] Arranz L, Fernandez C, Rodriguez A, Ribera JM, De la Fuente M (2008). The glutathione precursor N-acetylcysteine improves immune function in postmenopausal women. Free Radic Biol Med.

[35] Isola G, Lo Giudice A, Polizzi A, Alibrandi A, Murabito P, Indelicato F (2021). Identification of the different salivary Interleukin-6 profiles in patients with periodontitis: a cross-sectional study. Arch Oral Biol.

[36] Isola G, Polizzi A, Alibrandi A, Williams RC, Leonardi R (2021). Independent impact of periodontitis and cardiovascular disease on elevated soluble urokinase-type plasminogen activator receptor (suPAR) levels. J Periodontol.

[37] Isola G, Polizzi A, Alibrandi A, Williams RC, Lo Giudice A (2021). Analysis of galectin-3 levels as a source of coronary heart disease risk during periodontitis. J Periodont Res.

[38] Morawska K, Maciejczyk M, Zięba S, Popławski Ł, Kita-Popławska A, Krętowski J (2021). Cytokine/chemokine/growth factor profiles contribute to understanding the pathogenesis of the salivary gland dysfunction in euthyroid hashimoto’s thyroiditis patients. Mediators Inflamm.

[39] Hopkins RZ, Li RY. Essential of free radical biology and medicine. USA: Cell Med Press AIMSCI, Inc.; 2017.

[40] Martinez Banaclocha M, Martinez N (1999). N-acetylcysteine elicited increase in cytochrome c oxidase activity in mice synaptic mitochondria. Brain Res.

[41] Villani G, Attardi G (2000). In vivo control of respiration by cytochrome c oxidase in human cells. Free Radic Biol Med.

[42] Lushchak VL (2014). Classification of oxidative stress based on its intensity. Exp Clin Sci.

[43] Rushworth GF, Megson IL (2014). Existing and potential therapeutic uses for N-acetylcysteine: The need for conversion to intracellular glutahione for antioxidant benefits. Pharm Ther.

[44] Baumgardner JN, Shankar K, Hennings L, Albano E, Badger TM, Ronis MJ (2008). N-acetylcysteine attenuates progression of liver pathology in a rat model of nonalcoholic steatohepatitis. J Nutr.

[45] Martensson J, Lai JC, Meister A (1990). High-affinity transport of glutathione is part of a multicomponent system essential for mitochondrial function. Proc Nat Acad Sci USA.

[46] Kołodziej U, Maciejczyk M, Miąsko A, Matczuk J, Knaś M, Żukowski P (2017). Oxidative modification in the salivary glands of high fat-diet induced insulin resistant rats. Front Physiol.

[47] Ittichaicharoen J, Apaijai N, Tanajak P, Sa-Nguanmoo P, Chattipakorn N, Chattipakorn SC (2017). Impaired mitochondria and intracellular calcium transients in the salivary glands of obese rats. Appl Physiol Nutr Metab.

[48] Sato EF, Utsumi K, Inoue M (1996). Human oral neutrophils: isolation and characterization. Methods Enzymol.

[49] Liu M, Pelling JC, Ju J, Chu E, Brash DE (1998). Antioxidant action via p53-mediated apoptosis. Cancer Res.

[50] Bailey SM, Cunningham CC (1999). Effect of dietary fat on chronic ethanol-induced oxidative stress in hepatocytes. Alcohol Clin Exp Res.

[51] Kim H, Seo JY, Roh KH, Lim JW, Kim KH (2000). Suppression of NF-kappaB activation and cytokine production by N-acetylcysteine in pancreatic acinar cells. Free Radic Biol Med.

[52] Anrather J, Racchumi G, Iadecola C (2006). NF-kappaB regulates phagocytic NADPH oxidase by inducing the expression of gp91phox. J Biol Chem.

[53] Adamczyk-Sowa M, Bieszczad-Bedrejczuk E, Galiniak S, Rozmiłowska I, Czyżewski D, Bartosz G (2017). Oxidative modifications of blood serum proteins in myasthenia gravis. J Neuroimmunol.

[54] Zalewska A, Knaś M, Gińdzieńska-Sieśkiewicz E, Waszkiewicz N, Klimiuk A, Litwin K (2014). Salivary antioxidants in patients with systemic sclerosis. J Oral Pathol Med.

[55] Choromańska M, Klimiuk A, Kostecka-Sochoń P, Wilczyńska K, Kwiatkowski M, Okuniewska N, et al. Antioxidant defence, oxidative stress and oxidative damage in saliva, plasma and erythrocytes of dementia patients. Can salivary AGE be a marker of dementia? Int J Mol Sci. 2017, 18; 10.3390/ijms18102205 10.3390/ijms18102205 PMC5666885 29053628

[56] Maciejczyk M, Szulimowska J, Taranta-Janusz K, Werbel K, Wasilewska A, Zalewska A. Salivary FRAP as A Marker of Chronic Kidney Disease Progression in Children. Antioxidants (Basel, Switzerland). 2019;8; 10.3390/antiox8090409 10.3390/antiox8090409 PMC6769502 31540400

[57] Maciejczyk M, Szulimowska J, Skutnik A, Taranta-Janusz K, Wasilewska A, Wiśniewska N, et al. Salivary Biomarkers of Oxidative Stress in Children with Chronic Kidney Disease. J Clin Med. 2018;7; 10.3390/jcm7080209 10.3390/jcm7080209 PMC6111793 30103431

[58] Maciejczyk M, Mikoluc B, Pietrucha B, Heropolitanska-Pliszka E, Pac M, Motkowski R (2017). Oxidative stress, mitochondrial abnormalities and antioxidant defense in ataxia-teleangiectasia, bloom syndrome and nijmegen breakage syndrome. Redox Biol.

[59] Skutnik-Radziszewska A, Maciejczyk M, Fejfer K, Krahel J, Flisiak I, Kołodziej U (2020). Salivary antioxidants and oxidative stress in psoriatic patients: can salivary total oxidant status and oxidative stress index be a plaque psoriasis biomarker?. Oxid Med Cell Longev.

[60] Xiang RL, Huang Y, Zhang Y, Cong X, Zhang ZJ, Wu LL (2020). Type 2 diabetes-induced hyposalivation of the submandibular gland through PINK1/Parkin-mediated mitophagy. J Cell Physiol.

